# Healthcare Waste Management Practice and Associated Factors among Private and Public Hospitals of Bahir Dar City Administration

**DOI:** 10.1155/2020/7837564

**Published:** 2020-10-22

**Authors:** Dereje Mesfin Assemu, Tadese Ejigu Tafere, Yared Mulu Gelaw, Getasew Mulat Bantie

**Affiliations:** ^1^Bahir Dar City Zonal Health Department, Bahir Dar City, Ethiopia; ^2^Department of Health Economics and Health Service Management, School of Public Health Bahir Dar University, Bahir Dar City, Ethiopia; ^3^Department of Public Health, Alkan Health Science Business and Technology College, Bahir Dar City, Ethiopia

## Abstract

**Background:**

Lack of an appropriate management practice of healthcare waste is a potential threat to the healthcare workers, patients, and nearby communities of the health institutions.

**Objective:**

The study aimed to assess the healthcare waste management practices (HCWMP) and associated factors among healthcare workers of private and public hospitals of Bahir Dar city administration, Ethiopia.

**Methods:**

A facility-based comparative cross-sectional study was conducted from January 2016 to April 2017. The systematic random sampling technique was employed to recruit 460 healthcare workers. The collected data entered into the EpiData software (version 3.1). The analysis was done by using SPSS software (version 20). Descriptive statistics were computed; logistic regression model was run. The model fitness was checked using Hosmer and Lemeshow goodness of fit (*p* > 0.05). A *p* value of <0.2 at univariate analysis was included in the multivariable logistic regression analysis. Variables with a *p* value of <0.05 were statistically associated with healthcare waste management practice at 95% CI (AOR).

**Results:**

A total of 418 healthcare workers who participated in the study yielded a response of 90.9%. About 65% (95% CI: 61, 70) of the total respondents had good practice of healthcare waste management. More private hospitals, 79.2% (95% CI: 73, 85), had good healthcare waste management practice compared to public hospitals, 53.5% (95% CI: 47, 60). Male healthcare workers (AOR = 6. 43, 95% CI: 1.82, 22.77) and having a functional healthcare waste management committee (AOR = 6. 47, 95%CI: 1.93, 21.76) were significantly associated with HCWMP at private hospitals. For public hospitals, having a healthcare waste management committee (AOR = 1. 80, 95% CI: 1.03, 3.15) and a manual/guideline on HCWMP (AOR = 2. 43, 95% CI: 1.20, 4.91) was significantly associated with HCWMP.

**Conclusions:**

This study revealed there is a great discrepancy in HCWMP between private and public hospitals. Male healthcare workers and having a functional healthcare waste management committee and a manual/guideline were the identified factors of HCWMP.

## 1. Background

Healthcare facilities are one of the signiﬁcant sectors that have been showing improvement throughout the world during recent decades [1]. However, healthcare activities can lead to the generation of various types of waste that may have adverse effects on human health and on the environment [2].

Healthcare wastes are wastes produced by health service delivering facilities and laboratories [3]. These wastes are sharps, nonsharps, blood, body parts, chemicals, pharmaceuticals, medical devices, and radioactive materials [4]. Most of these are toxic, harmful, carcinogenic, and infectious materials [5, 6].

Healthcare wastes account for around 1-2% of urban wastes, which is a critical public health issue as they jeopardize human and environmental health [7]. Even though the current medical waste management practices vary from hospital to hospital, the awkward areas are similar to all health service delivering facilities [8]. Healthcare waste management survey in 22 developing countries revealed that the proportion of healthcare facilities that used inappropriate waste disposal method varies from 18% to 64% [9].

Improper healthcare waste management practice affects the healthcare workers, janitors, patients, and hospital environment [4, 10–13]. A proper healthcare waste management in a hospital depends on awareness, availability of training, personal protective equipment, dedicated waste management team, good administration, careful planning, sound organization, underpinning legislation, adequate financing, full participation by trained staff, use of appropriate disposal technique, and national regulatory framework [8, 14–20].

Proper healthcare waste management practice can prevent transmission of infectious diseases, environmental pollution, unpleasant smells, multiplication of insects, rodents, and worms [6, 17, 21]. However, healthcare waste management has not received enough attention in recent decades in economically developing countries [22]. Many findings in developing countries on healthcare wastes management revealed that segregation, collection, and storage of waste in isolated area were not satisfactory [18]; furthermore, healthcare wastes originating from healthcare facility dumped either into their backyard in a simple pit or put in open garbage to bins on the roads [23–26].

In Ethiopia, nowadays, healthcare facilities are becoming greater than ever to address the basic health needs of the community and to achieve the sustainable development goals (SDG). A few studies in Ethiopia revealed that there was no waste segregation in most studied healthcare facilities. But, they were stored, transported, treated, and disposed wrongly [26, 27].

The proper healthcare waste management practice thought the best strategy to halt the spread of the infectious disease. However, there is a scanty study of the healthcare waste management practice in Amhara region. Hence, determining the healthcare waste management level and identifying its determinants is important to understand the gap and strengthen the existing strategies. Though the healthcare waste management practice was low in Ethiopia, there is also an unclear discrepancy between the public and private healthcare facilities. Therefore, the purpose of this study was to determine and compare the healthcare waste management practice level between private and public hospitals and to identify its associated factors in Bahir Dar city hospitals.

## 2. Methods

### 2.1. Study Design Area and Period

The institution-based cross-sectional study was conducted in Bahir Dar city hospitals from January 2016 to April 2017. Bahir Dar city is the capital city of Amhara National, Regional State [28], and is located about 570 km northwest of Addis Ababa. The city is bordered by Lake Tana in the north, South Gondar in the east and by West Gojjam in the south and west. The city has 9 subcities and 12 rural kebeles with a total population of 308,887 [28]. The city has one university, two referral hospitals, one district hospital, ten health centers, and two private hospitals. The number of healthcare workers in both public and private facilities is 823. The study was conducted two public hospitals (one referral and one district) and two private hospitals.

### 2.2. Population

The source populations were all healthcare workers working in public and private hospitals in Bahir Dar city administration, while the study populations were selected healthcare workers in each healthcare facility working at least six months in the current hospital during the data collection period. Employed healthcare workers who are working less than six months in the current hospital were excluded.

### 2.3. Sample Size Determination

The sample size was calculated using the formula for two population proportions of the healthcare waste management practice level in public hospitals which is *p*1=40% [29] and in a private hospital is *p*2=55%, with 1 : 1 ratio between private and public hospitals, power = 80%, and confidence level = 95% (1.96). The formula can be seen as follows.

Accordingly, the required total sample size was 418. By considering a 10% nonresponse rate, the final sample size was 460 healthcare workers, 230 from private and 230 from public hospitals.

### 2.4. Sampling Procedures

**Figure 1:**
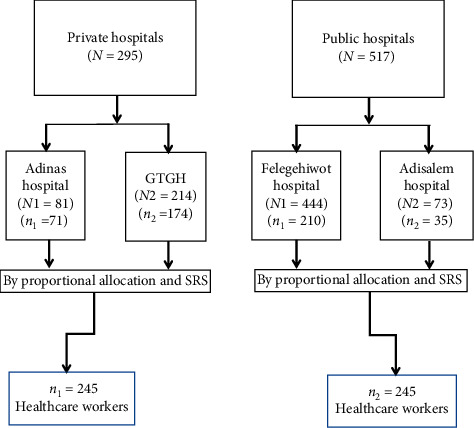
Schematic presentation of the sampling procedure on healthcare waste management, Bahir Dar city, 2017.

For this study, all private and public hospitals in Bahir Dar city administration were included. Proportional to size allocation was used to select the sample from each private and public hospital and from each type of healthcare workers. The respondents were stratified by their department and profession, and then the study participants were selected from each group using systematic random sampling. The study included selected healthcare workers who were on duty at the time of the data collection period (Figure 1).

### 2.5. Operational Definition

#### 2.5.1. Knowledge of Healthcare Waste Management

Healthcare workers who had a median score or above on the assessing questions regarding the knowledge of healthcare waste management were considered having a good knowledge of healthcare waste management. Otherwise, they were considered having poor knowledge of healthcare waste management.

#### 2.5.2. Healthcare Waste Management Practice

Healthcare workers who had a mean score or above on the assessing questions regarding the healthcare waste management practice were considered having good healthcare waste management practice. Otherwise, they were considered as having poor healthcare waste management practice [30].

### 2.6. Data Collection Procedure

Data were collected by using a structured interviewer-administered Amharic (indigenous language) questionnaire, which was adapted from different literature [31–33]. The questionnaire is comprised of sociodemographic, knowledge, and healthcare institution related characteristics. The questionnaire was developed in English and translated to Amharic and then back to English to keep the consistency. Pretest was done by 40 healthcare workers (20 from private and 20 from the public) in Bure town. A one-day training was given for four data collectors (having a bachelor in nursing) and two supervisors (having a bachelor in public health). At the end of every data collection day, the supervisors examined each questionnaire and gave pertinent feedback to the data collectors.

### 2.7. Data Processing and Analysis

The collected data were checked for completeness and consistency. The data were cleaned, coded, and entered into EpiData software. Analysis was done using SPSS (version 20) software. Descriptive statistics were computed. Simple logistic regression model was used to identify the association between the explanatory variables and HCWMP. Adjusted odds ratio (AOR) with 95% CI (confidence interval) was used to measure the strength of association between explanatory variables and the HCWMP. The model fitness was checked using Hosmer and Lemeshow goodness of fit (*p* > 0.05). A *p* value <0.2 at bivariate analysis was considered for variables to be included in multivariable analysis. Backward logistic regression method was used, and variables with a *p* value of <0.05 at multivariable analysis were considered as statistically significant predictors of HCWMP.

## 3. Results

### 3.1. Sociodemographic Information of Respondents

A total of 418 healthcare workers who are working in the private and public hospitals participated in the study and yielded a response of 90.9%. The overall mean age of the respondents was 28.81 (±7.29) years. About 35.9% of the private hospital healthcare workers were diploma holders, while 151 (36.1%) of the public hospital healthcare workers were degree graduate. More than one-third, 67 (34.9%), of the private hospital healthcare workers and majority of the public hospital healthcare workers, 123 (54.4%), were nurses. The majority of the private hospitals, 119 (62%), and public hospitals, 136 (60.2%), healthcare workers had a work experience of less than five years. More than two-thirds, 130 (67.7%), of the respondents were from GAMBY hospital, and more than three-fourth (85%) of the respondents were from Felegehiwot referral hospital (Table 1).

The study showed that 83 (43.2%) of private hospital and 100 (44.2%) of public hospital healthcare workers had training for the healthcare waste management, respectively. 161 (83.9%) of private hospital and 179 (79.2%) of public hospital healthcare workers had responded as there are the rules and regulations regarding healthcare waste management in their hospital. 133 (69.3%) of private hospital and 140 (61.9%) of public hospital healthcare workers had healthcare waste management team/committee (Table 2).

When assessing the knowledge level of healthcare workers regarding healthcare waste management practice using the composite index, 321 (72.2%) of healthcare workers had good knowledge of healthcare waste management practice. 147 (76.6%) of healthcare workers from private hospitals and 155 (68.6%) from public hospitals had good knowledge of the healthcare waste management (Table 3).

**Figure 2:**
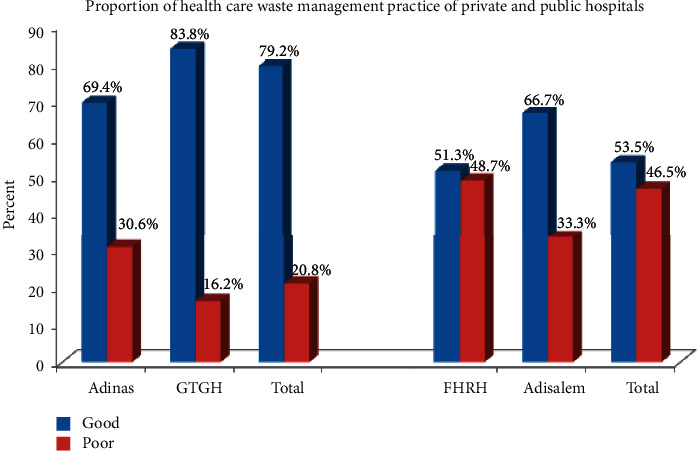
The proportion of healthcare waste management practice between private and public hospitals.

The overall good healthcare waste management practice was 65.3% (95% CI: 61, 70). More private healthcare workers, 79.2% (95% CI: 73, 85), had good healthcare waste management practice than public healthcare workers, 53.5% (95% CI: 47, 60), respectively (Figure 2).

### 3.2. Factors Associated with Healthcare Waste Management in Bahir Dar City Hospitals

Multivariable logistic regression analysis was conducted to identify independent predictors of healthcare waste management practice of Bahir Dar city hospitals. On univariate logistic regression model ownership of the hospital, having legislation/regulation in the hospital, reporting system related to waste handling injury/accident, healthcare waste management committee, good knowledge of healthcare waste management, and guideline/ manual regarding the healthcare waste management was associated with healthcare waste management practice at *p*-value of less than 0.2. However, in the multivariable logistic regression, healthcare facility ownership and having a healthcare waste management committee and a manual/guideline related to waste handling were significantly associated with healthcare waste management practice at *p* value of less than 0.05.

Accordingly, for healthcare workers working in a private hospital, the odds ratio of healthcare waste management practice was about two (AOR = 2.89, 95% CI: 1.85, 4.51) times higher compared to those working in public hospitals. Similarly, for healthcare workers who had a committee of healthcare waste management, the odds ratio of healthcare waste management practice was about 1.75 (AOR = 1 12, 95% CI: 1.12, 2.72) times higher compared to those who did not have one. It is also revealed that, in the hospitals which had a manual/guideline related to waste handling, the odds ratio of healthcare waste management practice was about two times (AOR = 2.06, 95% CI: 1.152, 3.684) higher compared to those who did not have one (Table 4).

Multivariable logistic regression analysis was conducted to identify independent predictors of healthcare waste management practice of Bahir Dar city private hospitals. In the univariate logistic regression, sex, having a healthcare waste management committee, functional healthcare waste management committee, reporting system related to waste handling injury/accident, and HCWMP included in the job description were associated with healthcare waste management practice at *p*-value of less than 0.2. However, in the multivariable logistic regression, only sex and having a functional healthcare waste management committee were significantly associated with healthcare waste management practice at *p*-value of less than 0.05.

For male healthcare workers working in a private hospital, the odds ratio of healthcare waste management practice was about six (AOR = 6.43, 95% CI: 1.82, 22.77) times higher compared to female healthcare workers. Similarly, for healthcare workers who have functional healthcare waste management committee, the odds ratio of healthcare waste management practice was about six (AOR = 6.47, 95% CI: 1.93, 21.76) times higher compared to those who did not have one (Table 5).

Multivariable logistic regression analysis was conducted to identify independent predictors of healthcare waste management practice of Bahir Dar city private hospitals. In the univariate logistic regression, sex, having rules and regulation/legislation on HCWM, a manual/guideline related to waste handling, and a healthcare waste management committee, and HCWMP included in the job description were associated with healthcare waste management practice at *p*-value of less than 0.2. However, in the multivariable logistic regression, only having a manual/guideline related to waste handling and healthcare waste management committee were significantly associated with healthcare waste management practice at *p*-value of less than 0.05.

Accordingly, for healthcare workers who had healthcare waste management committee, the odds ratio of healthcare waste management practice was about two (AOR = 1.80, 95%CI: 1.03, 3.15) times higher compared to those who did not have one. Similarly, for healthcare workers who have a manual/guideline related to waste handling, the odds ratio of healthcare waste management practice was about two (AOR = 2.43, 95%CI: 1.20, 4.91) times higher compared to those who did not have one (Table 6).

## 4. Discussion

The World Health Organization has organized a healthcare waste management guideline to warrant safe healthcare waste management. Furthermore, a Quality and Standard Authority of Ethiopia (in 2004) and the Federal Ministry of Health of Ethiopia (in 1997) had also prepared a guideline on healthcare waste handling and disposal within healthcare facilities [34].

This study determined the level of healthcare waste management practices among private and public hospitals and identified the associated factors in the Bahir Dar city. More private hospitals (79.2%) had a good healthcare waste management practice than public hospitals (53.5%). Male healthcare workers, having a functional healthcare waste management committee in private hospitals, and availability of healthcare waste management manual/guideline and committee in public hospitals were significantly associated with healthcare waste management practice.

The current study revealed that 79.2% (95% CI: 73, 85) of private hospitals had good healthcare waste management practice. The result of this study was higher than other studies on private hospitals in Addis Ababa, Ethiopia (40.48%) [35], Bahawalpur City, Pakistan (41.6%) [36], and Nigeria (62%) [37]. The possible justification for this discrepancy could be the variation of the study time. The former study was carried out recently, where the healthcare management practice is improving due to the increased awareness and attitude of the healthcare workers via IEC (behavioral change communication). The other possible justification for this variation could be using different healthcare waste management practice assessing tools across different studies.

The study also determined the healthcare waste management practice of public hospitals, which was 53.5% (95% CI: 47, 60). This finding was consistent with the findings of other studies on public hospitals in Addis Ababa 57.37% [38] (59.22%) [35] and Bangladesh (54%) [39]. However, the result of this study was lower than other studies done in Bahawalpur City, Pakistan (66.6%) [36] and Nigeria (98.4%) [40]. This might be due to the difference in healthcare system/policy and the healthcare planners towards the healthcare waste management practice. This finding might also be justified as a result of the ignorance of the healthcare workers regarding healthcare waste management practice.

In contrast, the result of the current study was higher than other studies on Adama Hospital, Ethiopia, 34.9% [41], Gondar Town, Ethiopia, 31.5% and 46.30% [42, 43], Nigeria, 42.1% [44], Burundi, 29.5% [45], and Bangladesh, 36.03% [46]. The possible reason for this variation could be the difference in the study period, study area, and sample size used.

The current study also identified various factors associated with healthcare waste management practices. Ownership of the hospital was also correlated with healthcare management practices. Healthcare workers working at private hospitals were more likely to be having good healthcare waste management practice than public hospital healthcare workers. This could be explained by the tendency that the private hospitals are more business-oriented and they need to attract the attention of the clients or patients with their clear and neat compound. They are also supposed to engage in the free market and should have the current standard equipment and this might allow good practice of healthcare waste management. Also, in the private hospitals, the community might expect safer and infection-free services as a result, and this might help them to best practice healthcare waste management.

This study has revealed that hospitals which had a healthcare waste management committee and manual/guideline related to healthcare waste handling had about 2-fold good healthcare waste management practice compared to those which did not have any. This finding was also observed in this study of public hospitals. This could be due to the fact that hospitals staff was advised on the importance of healthcare waste management practice by the committee members or by reading the manuals/guidelines of the healthcare waste management protocol.

Male sex was one of the explanatory variables statistically associated with good healthcare waste management practice at the private health facility, but not in the public health facilities.

The other significant predictor of healthcare waste management practice in private hospitals was having a functional healthcare waste management committee. Privately owned hospitals, which had functional healthcare waste management committee, were correlated with good healthcare waste management practices. However, no correlation was found at public hospitals. This might be due to the fact that healthcare workers in private hospitals have strict follow-up and supervision and evaluation programs, which in fact allow the formed committee to be well functional in practicing healthcare waste management, and as a result, private hospitals could have a better experience of healthcare waste management practice.

### 4.1. Strength and Limitation of the Study

Even though this study has provided valuable evidence regarding the level of healthcare waste management practice and the possible associated factors, it could not avoid the chicken-egg dilemma. There was also a challenge of having standard measuring cut-points for healthcare waste management practice. It was also difficult to collect the previously published articles related to healthcare waste management to make comparisons, particularly, in the currently identified predictors.

## 5. Conclusion

The study revealed that there was a great discrepancy between private and public hospitals in healthcare waste management practice. More private hospitals, 79.2% (95%CI: 73, 85), had a good healthcare waste management practice compared to public hospitals, 53.5% (95%CI: 47, 60). Male healthcare workers and having functional healthcare waste management committee were significantly associated with healthcare waste management practice in private hospitals, whereas having a healthcare waste management committee and a manual/guideline on healthcare waste management was significantly associated with healthcare waste management practice in public hospitals.

### 5.1. Recommendations

The Amhara Regional Health Bureau should do the following:

1. Focus on strategies regarding the healthcare waste management committee establishment in each hospital and the provision of enough healthcare waste management related manuals/guidelines to the hospitals
2. Organize short-term training and an orientation program on healthcare waste management for their staff

The private hospitals should do the following:

1. Share their best experiences on how they are practicing the healthcare waste management with the public hospitals
2. Train the public hospital healthcare workers on how the healthcare waste management committee is functioning
3. Train their female healthcare workers to have a better healthcare waste management practice

## Figures and Tables

**Table 1:** Sociodemographic characteristics of the respondents in Bahir Dar city hospitals, April 2017. Healthcare and institution related characteristics.

Variable	Private hospital		Public hospital		Total	
	Number	Percent	Number	Percent	Number	Percent
*Age (in years)*						
18–25	92	47.9	120	53.1	212	50.7
26–35	75	39.1	74	32.7	149	35.6
36–45	16	8.3	20	8.8	36	8.6
46 and above	9	4.7	12	5.3	21	5.0

*Sex*						
Male	90	46.9	94	41.6	184	44.0
Female	102	53.1	132	58.4	234	56.0

*Educational status*						
Secondary school and below	38	19.8	23	10.2	61	14.6
Certificate	6	3.1	1	.4	7	1.7
Diploma	69	35.9	74	32.7	143	34.2
Degree	48	25.0	103	45.6	151	36.1
Master	31	16.1	25	11.1	56	13.4

*Profession*						
Health officer	4	2.1	—	—	4	1.0
Physician	23	12.0	19	8.4	42	10.0
Laboratory	21	10.9	16	7.1	37	8.9
Pharmacist	18	9.4	17	7.5	35	8.4
Radiographer	7	3.6	7	3.1	14	3.3
Midwifery	8	4.2	16	7.1	24	5.7
Cleaner	33	17.2	23	10.2	56	13.4
Environmental health officer	2	1.0	2	.9	4	1.0
Nurse	67	34.9	123	54.4	190	45.5
Laundry workers	9	4.7	3	1.3	12	2.9

*Work experience (in years)*						
0–5	119	62.0	136	60.2	255	61.0
6–10	41	21.4	60	26.5	101	24.2
≥11	32	16.7	30	13.3	62	14.8

*Respondents affiliation (hospital)*						
Adinas	62	32.3	—	—	62	14.8
GAMBY	130	67.7	—	—	130	31.1
Felegehiwot	—	—	193	85.4	193	46.2
Addisalem	—	—	33	14.6	33	7.9

**Table 2:** Healthcare and institution related characteristics in public and private hospitals in Bahir Dar city, April 2017. Knowledge-related characteristics.

Variables	Private hospitals		Public hospitals		Total	
	Number	Percent	Number	Percent	Number	Percent
*Have you taken any training on healthcare waste management?*						
Yes	83	43.2	100	44.2	183	43.8
No	109	56.8	126	55.8	235	56.2

*Are there any healthcare waste management rules and regulations in your facility?*						
Yes	161	83.9	179	79.2	340	81.3
No	12	6.3	16	7.1	28	6.7
Do not know	19	9.9	31	13.7	50	12.0

*Does your healthcare facility have healthcare waste management system?*						
Yes	103	53.6	113	50.0	216	51.7
No	89	46.4	113	50.0	202	48.3

*Does your healthcare facility have a waste management team/committee?*						
Yes	133	69.3	140	61.9	273	65.3
No	59	30.7	86	38.1	145	34.7

*Is your healthcare facility waste management team functional?*						
Yes	91	68.4	84	60.0	175	64.1
No	42	31.6	56	40.0	98	35.9

*Is there any guideline document on healthcare waste management you are aware of?*						
Yes	172	89.6	194	85.8	366	87.6
No	20	10.4	32	14.2	52	12.4

*Is there any regular healthcare waste management training for all staff?*						
Yes	23	12.0	31	13.7	54	12.9
No	169	88.0	195	86.3	364	87.1

*Is there enough personal protective equipment and other supplies for HCWM?*						
Yes	134	69.8	151	66.8	285	68.2
No	58	30.2	75	33.2	133	31.8

*Have you ever experienced waste handling related injury in the last 12 months?*						
Yes	23	12.0	31	13.7	54	12.9
No	169	88.0	195	86.3	364	87.1

**Table 3:** Knowledge-related characteristics in public and private hospitals in Bahir Dar city, April 2017. The prevalence of healthcare waste management practice between private and public hospitals.

Variables	Private		Government		Total	
	Number	Percent	Number	Percent	Number	Percent
*Improper color-coding segregation of healthcare waste increases the risk of injury*						
Yes	188	97.9	220	97.3	188	97.9
No	4	2.1	6	2.7	4	2.1

*Improper color-coding segregation of healthcare waste increases the amount of infectious healthcare waste*						
Yes	184	95.8	217	96.0	401	95.9
No	8	4.2	9	4.0	17	4.1

*Improper color-coding segregation of healthcare waste contributes to improper healthcare waste disposal*						
Yes	155	80.7	169	74.8	324	77.5
No	37	19.3	57	25.2	94	22.5

*Mixing of infectious waste with noninfectious waste contributes to disease transmission*						
Yes	147	76.6	171	75.7	318	76.1
No	45	23.4	55	24.3	100	23.9

*Improper healthcare waste disposal contributes to disease transmission*						
Yes	177	92.2	220	97.3	397	95.0
No	15	7.8	6	2.7	21	5.0

*Disposing of HCW without treatment contributes to disease transmission*						
Yes	28	14.6	42	18.6	70	16.7
No	164	85.4	184	81.4	348	83.3

*HIV can be transmitted from unsafe healthcare waste management practice*						
Yes	179	93.2	211	93.4	390	93.3
No	13	6.8	15	6.6	28	6.7

*Hepatitis B and C can be transmitted from unsafe healthcare waste management practice*						
Yes	171	89.1	193	85.4	364	87.1
No	21	10.9	33	14.6	54	12.9

*Hepatitis A can be transmitted from unsafe healthcare waste management practice*						
Yes	140	72.9	128	56.6	268	64.1
No	52	27.1	98	43.4	150	35.9

*Ebola can be transmitted from unsafe healthcare waste management practice*						
Yes	159	82.8	162	71.7	321	76.8
No	33	17.2	64	28.3	97	23.2

*Knowledge score*						
Good	147	76.6	155	68.6	302	72.2
Poor	45	23.4	71	31.4	116	27.8

**Table 4:** Factors associated with healthcare waste management (HCWM) practice in Bahir Dar city hospitals, 2017 (*n* = 418). Factors associated with healthcare waste management in private hospitals.

Variables	HCWM practice		COR at 95% CI	AOR at 95% CI	*p* value
	Good	Poor			
*Ownership of the hospital*					
Private	152	40	3.298 (2.133, 5.097)	**2.89 (1.85, 4.51)**	**0.0001**
Public	121	105	1.00	1.00	

*Rules and regulations/legislations on HCWM in your facility*					
Yes	231	109	1.817 (1.102, 2.995)	1.28 (0.702, 2.326)	0.423
No	42	36	1.00	1.00	

*Healthcare waste management committee*					
Yes	193	80	1.96 (1.29, 2.978)	**1.75 (1.123, 2.72)**	**0.013**
No	80	65	1.00	1.00	

*Manual/guideline related to waste handling*					
Yes	242	111	2.39 (1.39, 4.087)	**2.06 (1.152, 3.684)**	**0.015**
No	31	34	1.00	1.00	

*Having a reporting system related to waste handling injury/accident*					
Yes	180	82	1.59 (1.05, 2.41)	0.89 (0.532, 1.508)	0.678
No	87	63	1.00	1.00	

*Knowledge of healthcare waste management*					
Good	207	95	1.651(1.06, 2.56)	1.315 (0.814, 2.123)	0.263
Poor	66	50	1.00	1.00	

**Table 5:** Factors associated with healthcare waste management (HCWM) practice in Bahir Dar city private hospitals, 2017 (*n* = 192). Factors associated with healthcare waste management in public hospitals.

Variables	HCWM practice		COR at 95% CI	AOR at 95% CI	*p* value
	Good	Poor			
*Sex*					
Male	74	15	1.623 (1.794, 3.318)	**6.43 (1.817, 22.769)**	**0.004**
Female	77	25	1.00	1.00	

*Having a healthcare waste management committee*					
Yes	109	24	1.690 (1.819, 3.487)	1.32 (0.23, 2.72)	0.58
No	43	16	1.00	1.00	

*Having a functional healthcare waste management committee*					
Yes	69	11	2.039 (1.235, 4.976)	**6.473 (1.93, 21.76)**	**0.003**
No	40	13	1.00	1.00	

*Having a reporting system related to waste handling injury/accident*					
Yes	105	26	1.379 (1.656, 2.900)	0.524 (0.124, 2.217)	0.380
No	41	14	1.00	1.00	

*Healthcare waste management included in the healthcare worker's job description*					
Yes	89	25	1.903 (1.37, 3.867)	1.018 (0.306, 3.380)	0.977
No	57	15	1.00	1.00	

**Table 6:** Factors associated with healthcare waste management (HCWM) practice in Bahir Dar city public hospitals, 2017 (*n* = 226).

Variables	HCWM practice		COR at 95% CI	AOR at 95% CI	*p* value
	Good	Poor			
*Sex*					
Male	43	51	0.584 (0.342, 0.996)	0.668 (0.384, 1.160)	0.152
Female	78	54	1.00	1.00	

*Availability of rules and regulations/legislations on HCWM in your facility*					
Yes	104	75	2.447 (1.259, 4.758)	1.276 (0.562, 2.897)	0.561
No	17	30	1.00	1.00	

*Availability of a manual/guideline related to waste handling*					
Yes	106	75	2.827 (1.422, 5.617)	**2.430 (1.203, 4.908)**	**0.013**
No	15	30	1.00	1.00	

*Availability of a healthcare waste management committee*					
Yes	84	56	1.986 (1.152, 3.424)	**1.801 (1.029, 3.152)**	**0.039**
No	37	49	1.00	1.00	

*Healthcare waste management included in the job description*					
Yes	86	56	2.106 (1.215, 3.652)	1.363 (0.723, 2.569)	0.338
No	35	48	1.00	1.00	

## Data Availability

The data are available from the corresponding author upon justified request.

## Abbreviations

AOR: Adjusted odds ratio
BSc: Bachelor of science
COR: Crude odds ratio
EpiData: Epidemiological data
IEC: Information Education and Communication
MOH: Ministry of Health
MPH: Master of public health
SPSS: Statistical Package for Social Sciences.
